# Efficacy of* Achyranthes aspera* (L.) as a Molluscicidal Bait Formulation against Fresh Water Snail* Biomphalaria pfeifferi*

**DOI:** 10.1155/2018/2718585

**Published:** 2018-06-27

**Authors:** Belayhun Mandefro, Seid Tiku Mereta, Argaw Ambelu

**Affiliations:** ^1^Department of Environmental Health Science and Technology, Faculty of Public Health, Jimma University, P.O. Box 378, Jimma, Ethiopia; ^2^Department of Biology, College of Natural and Computational Sciences, Dilla University, P.O. Box 419, Dilla, Ethiopia

## Abstract

Molluscicidal plant products have interesting attributes of environmental friendliness and accessibility to local communities. Their bait formulations are more economical and target specific as they are applied only to the snail-infested sections of the water habitat. Their active ingestion by target snails could also increase gastric concentrations and enhance effectiveness. This study aimed to evaluate the comparative effectiveness of* Achyranthes aspera* (*A. aspera*) leaf hydroethanolic extract in bait and immersion applications. Serial dilutions of the extract in water for immersion, and in snail food pellets for bait test, were set. Adult* Biomphalaria pfeifferi *snails exposed to the bioassays for 24 hours and data were analyzed using probit model. The plant showed molluscicidal activity in both methods. The respective LC_50_ and LC_90_ doses were 20.37 and 46.84 ppm in the immersion and 3.10 and 11.08 ppm in the bait. The more efficient bait method reduced the LC_50_ by 6.57 and the LC_90_ by 4.23 times. This finding provides a strong foundation for the molluscicidal potential of* A. aspera*. It is applicable and possibly more effective if formulated into those commercially available snail food pellets or flakes. However, selection and optimization of suitable baits is a crucial step for maximum output.

## 1. Background

Schistosomiasis control can be more successful when chemotherapy and snail control activities are integrated [1, 2]. Recently, molluscicidal plants are drawing more and more attention for their environmental friendliness, accessibility, and cost effectiveness. They are especially suitable for community based snail control activities in places where schistosomiasis transmission is more focal [3]. So far, several molluscicidal plants have been screened worldwide.* Carica papaya* (*C*.* papaya*),* Terminalia catappa *(*T. catappa*) [4] and* Solanum *species [5] are just some of them.* Glinus lotoides *(*G. lotoides*) [6], and* Pueraria peduncularis *(*P. peduncularis*) [7] are also among the recently discovered ones.

The relatively low efficacy of many molluscicidal plants as compared to niclosamide is a hindrance to their practical applicability [8]. Parallel to the search for noble molluscicides of higher efficacy and safety, adoption of efficient application mechanisms could play a decisive role in enhancing their effectiveness. In principle, such molluscicides should be applied in dose as low as possible for environmental, natural resource conservation and economic reasons [9, 10]. In the usual immersion method, the molluscicide is added to the water habitat [11, 12] and is needed in large amount to titrate the entire water body. Alternatively, molluscicidal baits are formulated irrespective of the water volume and are applied only to the snail-infested sections requiring only a very small amount of active ingredients. Baits use as carriers of the molluscicide and attractants which increase palatability of the substance by target snails [13–15]. Such baits have been practiced against freshwater snail* Lymnaea acuminata *by some researchers [16–18].

*Achyranthes aspera* (*A. aspera*) is a perineal herb in the family Amaranthaceae. The plant grows in many parts of Ethiopia on roadsides, on wastelands, near hedges, and under eucalyptus tree plantations. It is a known medicinal plant in Ethiopian folklore used for placental retention, postpartum bleeding, skin eruptions, and wound dressing [19, 20]. This study is aimed to evaluate the comparative efficacy of* A. aspera* leaf hydroethanolic extract against* Biomphalaria pfeifferi *(*B. pfeifferi*) snails in molluscicidal bait and immersion applications, in the laboratory condition.

## 2. Material and Methods

### 2.1. Plant Material Collection and Processing

The plant was selected for this study based on the researchers' previous screening tests. Mature green leaves were collected in October 2016 from a natural habitat located at 9°43′45.59′′ N, 39°73′2.71′′ E, central Ethiopia. It was dried in the shade and ground to fine powder of 200 *μ*m mesh size as in Adenusi and Odaibo [21]. It was stored in plastic bags in the laboratory of Environmental Health Science and Technology, Jimma University, Ethiopia. Experts in Addis Ababa University Herbarium have identified and authenticated the plant species. Voucher specimen with specimen number: M.B.1 is kept in the herbarium.

### 2.2. Extract Preparation

Considering the relative polarity of the presumed molluscicidal phytochemicals such as saponins [1, 22], hydroethanol is selected as an efficient extraction solvent. In addition, hydroethanol renders more extract yield than absolute ethanol [22, 23]. Ethanol is also safer and less toxic than methanol, acetone, and other organic solvents. Extraction was done with slight modification of the procedure in Ndamukong and colleagues [24]. Exactly 100 g plant powder was rinsed in 1000 ml of 80% ethanol in a flat-bottomed airtight flask and shook on an orbital shaker for 24 hours at 125 rpm at room temperature. After filtration with Whatman filter paper (110 mm thickness CAT No. 1540 110), the filtrate evaporated from a wide mouth beaker placed in a water bath at 40°C. The resulting blue-black sticky amorphous matrix was stored in a dry clean container.

### 2.3. Phytochemical Analysis

Qualitative phytochemical screening tests were done on the extract based on standard procedures described in Akinyemi et al. [25], Sasidharan et al. [26], and Mungenge et al. [27]. Reducing sugars were identified by Fehling's test. Biuret test was used for proteins, froth test for saponins, 5% ferric chloride for total phenolics, and 10% ferric chloride and gelatin test for tannins. Alkaline test and concentrated sulphuric acid in ammonia were used for flavonoid test. In addition, Wagner's test for alkaloids, Salkowski reaction for terpenoids, and Liebermann-Burchard test for steroids were applied.

### 2.4. Snail Food Preparation

We used lettuce (*Lactuca sativa*) leaves for snail food. About 2 kg of young leaves, separated from the stems and midribs, was partially cooked in boiling tap water for 2 minutes. The par-boiled leaves were dried under the shade on a clean plastic sheet. It was ground to a fine powder of 250 *μ*m mesh size and stored in airtight plastic bags.

### 2.5. Snail Collection and Maintenance

We collected adult snails of* B. pfeifferi *from stream habitats located at 7°41′15.39′′ N, 36°50′52.32′′ E, in Jimma Town Public Prison agricultural field, southwestern Ethiopia. Snails were transported to the laboratory in clean plastic buckets half-filled with water from the streams. We acclimatized them in the laboratory for one week in 3 clean large plastic buckets filled with 10 l aged water, each containing 200 snails, at room temperature under 12 h light and 12 h dark photoperiods. Snails were fed with the previously prepared lettuce leaf powder by spreading about 0.25 g powder into each bucket in every 24 hours and water was replaced in every 3 days. The snails were ethically handled throughout the experiment according to the principles and guidelines of animal welfare in scientific researches [28, 29]. They were not subjected to cercaria shedding before or after the experiment. However, an independent and contemporary study indicated a 28.5% snail infection rate (Bedewi et al., unpublished).

### 2.6. Molluscicidal Test Assay in Immersion Method

The molluscicidal test assay was established based on standard procedures adopted for immersion type of test [30]. Stock solution of 1000 ppm was prepared by dissolving 0.5 g plant extract in 500 ml aged water. From this, 100 ml dilutions of 3.13, 6.25, 12.5, 25, 50, and 100 ppm were prepared in clean beakers. Ten adult* B. pfeifferi* snails, shell diameter 9.5-10.6 mm, were exposed to each dilution without food for 24 hours at room temperature. The test was performed in three replicates. Positive control was prepared from 1.5 ppm niclosamide and negative control from only aged water. After 24 hours, the snails in their respective groups were removed from the solutions, washed with aged water in a childcare manner, and transferred to new beakers with aged water and food for another 24 hours. Finally, dead and alive snails were counted through careful inspection. Snails were considered dead if they lose sense when the foot is pocked with needle or if they remain retracted in to the shell or else the foot is discolored [6, 30].

### 2.7. Molluscicidal Test Assay in Bait Method

Molluscicidal baits in the form of pellets were prepared based on the methods in Laskowski and Hopkin [31] and Srivastava et al. [18]. Different masses of the extract, 0.024, 0.047, 0.094, 0.188, 0.375, 0.750, and 1.500 mg, dissolve in 10 ml distilled water. To each of these solutions, a 5 g lettuce powder was added to make a 15 g homogenized pellet. Hence, the corresponding concentrations of the extract in the pellets were 1.57, 3.13, 6.25, 12.5, 25, and 50 ppm. Each pellet was evenly pasted on the inside base of a clean Petri dish (9.5 mm diameter) by pressing with smooth flat wooden applicator. Ten adult* B. pfeifferi* snails of 9.5-10.8 mm shell diameter were released in to each Petri dish for 24 hours. Wet cotton was put over the snails to maintain wet environment as adapted from Laskowski and Hopkin [31]. The test was carried out in four replicates with positive control made from pellet consisting of 1.5 ppm niclosamide (Bayer and Pro-Serv. Inc. Germany) and negative control pellet from only lettuce powder. After 24 hours, the snails from each bait were collected, carefully washed, and maintained in clean beakers with aged water and food for another 24 hours. Finally, live and dead snails in each treatment group were counted with careful inspection. Snail death was confirmed by loss of sense when pocked with a needle or by discolored mantle.

### 2.8. Data Analysis

The mortality data were analyzed using probit regression model in IBM SPSS program version 23 to determine the LC_50_ and LC_90_ doses with their respective upper and lower limits [32, 33]. These are the principal endpoints considered as a measure of molluscicidal efficacy. In addition, the nature of correlations existing between concentrations and mortality rates were assessed from the slope function.

## 3. Results

### 3.1. Phytochemical Analysis of the Leaf Hydroethanolic Extract

The phytochemical screening test results, (Table 1) revealed that the extract consists of a number of bioactive secondary metabolites. Saponins and flavonoids were found in relative abundance but tannins and proteins were not detected in this particular test.

### 3.2. Molluscicidal Activity

In the immersion test, snails exhibited irritative behavior and try to escape from the solutions by crawling up beyond the water level. They produced more mucus. Bleeding was observed later in the course of the experiment. They started to show signs of paralysis or death after the first six hours of exposure. When snails were exposed to the molluscicidal baits, they come out of their shells and move about over the pellet in the first hour. Then they gradually became inactive, reluctant to move, and started retracting to their shells. During the rehabilitation period, survivors come out of their shells and resumed activity.

Snail mortalities in the two tests are presented in Table 2. The record clearly showed that mortality rates increase with increasing concentrations of the extract.

The resulting probit analysis (Table 3) shows that the calculated *χ*^2^ values are less than the tabular values. This indicates that the data are qualified for Pearson's goodness of fit. In addition, the positive slope indicates positive correlations between mortality rates and concentrations.

## 4. Discussion

The findings of this study revealed* A. aspera* has a concentration-dependent molluscicidal effect against adult* B. pfeifferi *snails in both immersion and bait applications. However, the two methods exhibited different efficacies. The LC_50_ concentration in the bait is smaller than that in the immersion test (Table 3). This indicates the plant is more efficacious when applied as molluscicidal bait than its immersion form.

Previous studies have shown that the aqueous extract of* A. aspera* is effective against* B. pfeifferi* snails with 72.4 ppm LC_50_ and 96.5 ppm LC_90_ [34]. In the current study, the hydroethanolic extract exhibited a higher potency (20.37 ppm LC_50_ and 46.84 ppm LC_90_) by the same immersion technique. Similar to many other studies [35], the alcoholic extract is more potent than the aqueous one. Ethanol consists of both polar and nonpolar terminals at its opposite ends. Therefore it is capable of dissolving and extracting multitudes of organic compounds with a range of polarity.

Phytochemical analysis of this particular extract indicated presence of saponins, flavonoids, alkaloids, and total phenolics. Thus, the molluscicidal property of this plant is due to the availability of these bioactive phytoconstituents [1, 36]. Saponins are important molluscicidal compounds that disrupt cellular permeability and osmoregulatory functions by interacting with membrane sterols of cells [5]. Alkaloids and terpenoids mainly act as acetylcholinesterase inhibitors resulting in neurotoxicity in snails [30, 37].

There are studies regarding the toxicological and pharmacological aspects of* A. aspera*. Many researches investigated no significant mammalian toxicity of the plant [38, 39]. Rather, its antioxidant and anti-inflammatory properties due to the presence of saponins, alkaloids, and oleanolic acid [40, 41] are well known. On the other hand, antifertility and spermicidal properties [42, 43] as well as larvicidal property against* Ae. aegypti* [44] are indicated.

Several plants have exhibited molluscicidal activity against* B. pfeifferi* snails and related species. In the current study, the LC_50_ from hydroethanolic extract of* A. aspera *leaf is 20.37 ppm. It is proved to be more potent than* Cymbopogon citratus *where the LC_50_ is 159.47ppm [45]. It is also more potent than* G. lotoides* aqueous and ethyl acetate extracts in Kiros et al. [6] as well as* Entada leptostachya *and* Azadirachta indica *methanol extracts in Michael and colleagues [46].

**Figure 1:**
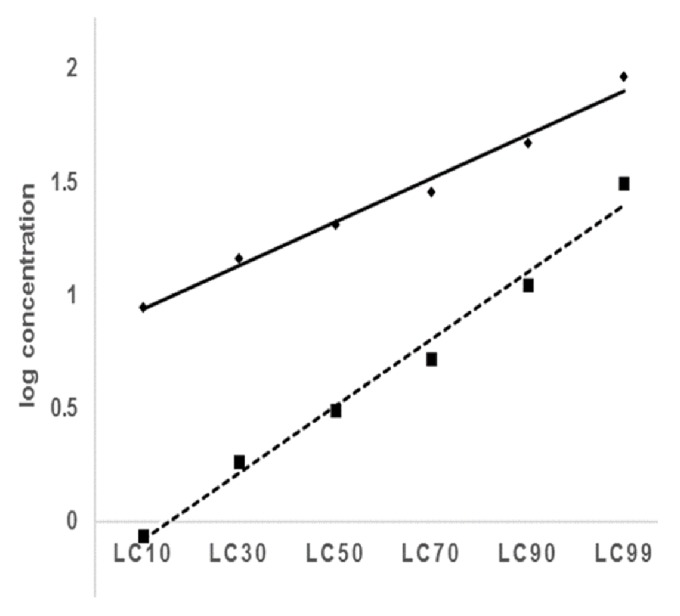
Comparative molluscicidal efficacy of* A. aspera* leaf hydroethanolic extract in bait and immersion tests against* B. pfeifferi* adult snails (broken line represents the bait test; solid line represents the immersion test).

The current study also showed a higher molluscicidal efficacy of* A. aspera *in the bait application as compared to the ordinary immersion method (Table 3). The bait application reduced the LC_50_ and LC_90_ lethal concentrations by about 6.6 and 4.2 times, respectively. The enhancement on the LC_50_ is greater than that on the LC_90_. Generally, greater efficacy enhancement was achieved in the sublethal doses than in the higher doses (Figure 1). According to previous studies, higher molluscicide titers could alter the natural taste and attractant quality of the snail food. Snails may hesitate to consume too much of it, hence reducing its palatability and effectiveness.

In line with this result, some studies have reported enhanced molluscicidal effectiveness in bait formulations [16, 17]. Nevertheless, bait effectiveness is a function of several factors such as palatability, attractant property, snail life stages, and many others [14, 17, 47]. The present study implies that alternative molluscicide applications such as baits with appropriate snail foods and attractants can be one possible mechanism to enhance the efficacy.

In immersion application, the molluscicide action is mainly by contact poisoning of the epithelial tissues in the head-foot region. Different studies reported closing of operculum, increased mucus secretion, and retracting into the shell during immersion [48]. Such protective behaviors can prevent free imbibition of molluscicidal compounds into the inner body cavities. On the other hand, molluscicidal baits act as stomach poisons [49]. Hence damage to the gastric epithelium is the main mechanism of action [14, 50, 51]. Increased efficacy in molluscicidal bait application can also be associated with other related factors. If snails avidly ingest the bioactive compounds with the food, they may accumulate in the gut to a higher local concentration at least temporarily before excretion. As the local concentration increases, greater damage is certainly expected. On the other hand, the gastric epithelium provides a larger area of poison contact than the cephalopodal surface. It may also facilitate the compounds' dispersal to hepatic tissues and other vital organs.

Molluscicidal bait application in the control of aquatic snails has additional advantages. It prevents chemicals from direct dispersal in to the aquatic environment. Attractants in the bait lure target snails so that they ingest the molluscicide actively. On the other hand, since the bait is applied only to the snail-infested section of the water habitat, it is more economical and target specific [16, 52].

## 5. Conclusion

Molluscicide application in the field needs several considerations. It should be applied in lowest possible dose to minimize interference with the physicochemical properties of the aquatic habitats and adverse effects on nontarget organisms. It should also be economical for sustainable use of those valuable medicinal plants. Therefore, selection of plants with better molluscicidal efficacy and safety is mandatory. Besides, modified application techniques that enhance their effectiveness are equally important.

This study confirms the molluscicidal activity of* A. aspera *from hydroethanolic extract of the leaf part in both immersion and bait application techniques. The findings provide a strong foundation to the molluscicidal potential of this plant for community based snail control activities. Furthermore, an appreciably higher efficacy resulted from the bait test indicating possibility of its application by formulating into those commercially available snail food pellets or flakes. The research further indicated that bait applications could be one possible mechanism to enhance molluscicidal efficacy of many claimed plants as long as appropriate foods and attractants are used.

## Figures and Tables

**Table 1:** Results of phytochemical screening tests on *A. aspera* leaf hydroethanolic extract. Here their relative abundances are expressed as +++ for more abundant, ++ for moderately abundant, + for trace, and – for none.

Phytoconstituents	Screening tests	Observation
reducing sugars	Fehling's test	+
proteins	Biuret's test	-
saponins	froth/foam test	+++
total phenolics	5% FeCl_3_ test	++
tannins	10% FeCl_3_ test	-
	gelatin test	-
flavonoids	reaction with H_2_SO_4_ in ammonia	+++
	alkaline test (KOH)	-
alkaloids	Wagner's test	++
terpenoids	Salkowski reaction	+
steroids	Liebermann-Burchard reaction	+

**Table 2:** Mortality data showing the total number of exposed and dead *B. pfeifferi* snails in each concentration of *A. aspera* leaf hydroethanolic extract during immersion and bait tests.

Immersion test			Bait test		
concentration (ppm)	exposed	dead	concentration (ppm)	exposed	dead
0.00	30	0	0.00	40	2
3.13	30	0	1.57	40	7
6.25	30	2	3.13	40	23
12.50	30	7	6.25	40	32
25.00	30	15	12.50	40	37
50.00	30	29	25.00	40	38
100.00	30	30	50.00	40	40

**Table 3:** The LC_50_ and LC_90_ doses (with confidence limits) of *A. aspera* leaf hydroethanolic extract against *B. pfeifferi* snails in immersion and bait tests.

Test method	Effective doses	Confidence limits	*χ* ^2^ (95% CL)	Slope
Immersion	LC_50_ = 20.37	16.97-24.57	4.158	3.544
	LC_90_ = 46.84	36.86-67.12		
Bait	LC_50_ = 3.10	1.29-5.16	4.760	2.319
	LC_90_ = 11.08	7.04-17.00		

## Data Availability

All data required for the analysis and conclusion are included in this article.
